# Predicting In-Hospital Cardiac Arrest Using Machine Learning Models: Protocol for a Scoping Review

**DOI:** 10.2196/69716

**Published:** 2025-09-09

**Authors:** Mina Attin, Bryar Shareef, Nelson Appiah-Agyei, Farzana Mahamud Rini, Xan Goodman, Lauren Bredesky, Jonathan A Chavez, Rawa Mohammed, Kavita Batra

**Affiliations:** 1 University of Nevada, Las Vegas Las Vegas, NV United States

**Keywords:** machine learning, electronic health records, artificial intelligence, AI, cardiac arrest, predictive value, resuscitation

## Abstract

**Background:**

In-hospital cardiac arrest (IHCA) remains a public health conundrum with high morbidity and mortality rates. While early identification of high-risk patients could enable preventive interventions and improve survival, evidence on the effectiveness of current prediction methods remains inconclusive. Limited research exists on patients’ prearrest pathophysiological status and predictive and prognostic factors of IHCA, highlighting the need for a comprehensive synthesis of predictive methodologies.

**Objective:**

This scoping review aims to synthesize and critically evaluate the quality and quantity of clinical features and machine learning (ML) models for predicting IHCA. The review will evaluate temporal characteristics, predictive and prognostic values of prearrest clinical features, and model performance metrics.

**Methods:**

This scoping review follows the PRISMA-ScR (Preferred Reporting Items for Systematic Reviews and Meta-Analyses extension for Scoping Reviews) guidelines and aims to synthesize studies that used ML algorithms to predict IHCA published between April 2009 and April 2024. We will conduct a comprehensive search using 4 major databases: PubMed, Web of Science, IEEE Xplore, and Embase. The inclusion criteria are peer-reviewed, English-language studies that explore ML applications for predicting IHCA in adult patients (aged ≥18 years). Exclusion criteria include review articles, preprints, non–English-language studies, and studies without specific ML metrics for IHCA prediction. Two independent reviewers will conduct the screening and data extraction using Rayyan for deduplication and ensuring study eligibility. Descriptive statistics will be used to summarize the data, and a narrative synthesis will provide insights into the clinical features used in the models, the performance metrics, and any gaps in the literature.

**Results:**

A total of 2479 records were identified between April 2009-April 2024. After removing duplicates and conducting screening, 16 studies have been included in the review. Data extraction and synthesis are ongoing and are expected to be completed by June 2025. The anticipated results from this review will provide a comprehensive overview of the clinical predictors of IHCA used in ML models, including commonly reported clinical features such as vital signs, biomarkers, and comorbidities. We expect to highlight variations in data quality and quantity across studies, which may influence model performance.

**Conclusions:**

This study will contribute to advancing ML applications for IHCA prediction by addressing data challenges and promoting standardization to improve the clinical decision-making process. The results of this review are expected to inform future studies; promote consistency in the reporting of clinical features; and, ultimately, enhance the decision-making process in clinical settings, potentially leading to better outcomes for patients experiencing IHCA.

**International Registered Report Identifier (IRRID):**

DERR1-10.2196/69716

## Introduction

In-hospital cardiac arrest (IHCA)—defined as the absence of a pulse and need for defibrillator shocks and/or chest compression in a patient admitted to an inpatient bed—remains a significant public health challenge in the United States, with approximately 292,000 cases occurring annually [1-3]. Mortality rates for IHCA are 80% for patients with nonshockable rhythms and 55% for those with shockable rhythms, and 1-year survival after IHCA is only 13% [2,4,5]. Despite documented differences between IHCA and out-of-hospital cardiac arrest (OHCA) [6], treatment guidelines for the 2 types remain the same. IHCA has been less studied compared to OHCA, with a recent study reporting 61 published randomized controlled trials involving OHCA compared to only 15 for IHCA (2015-2022) [7]. Most IHCA studies have focused on patient outcomes during or after resuscitation, with limited research into the complex prearrest pathophysiological changes. However, recent evidence has challenged traditional assumptions about the causes of IHCA (eg, hypoxia). Conditions such as sepsis or infection and heart failure, which lead to circulatory and respiratory collapse, have been identified as significant contributors that enhance our understanding of IHCA causes [8]. A deeper understanding of the complex pathophysiological changes and factors contributing to IHCA would enable the development of more accurate tools, early initiation of preventive measures, and more effective treatment strategies.

Early identification of high-risk patients could facilitate the implementation of preventive interventions to reduce mortality and potentially improve 1-year survival rates. However, evidence regarding the effectiveness of current prediction methods (eg, scoring systems) and the establishment of practices for preventing IHCA remains inconclusive [9,10]. Heterogeneity in settings; subgroups, including small sample sizes (eg, patients with cardiac arrest); and methodologies, including lack of high-quality validation in common diseases that cause mortality (eg, cardiovascular diseases), as well as insufficient reporting, make the scoring system suboptimal to predict patient deterioration and cardiac arrest [9,11-13]. Moreover, the effective role of rapid response teams (RRTs) in preventing IHCA remains controversial. A crucial factor in preventing IHCA is the identification of deteriorating signs by the clinicians and activation of RRTs. Delayed activation or failure to activate RRTs has been associated with increased mortality [14]. Furthermore, a study by the investigators of the American Heart Association’s Get With the Guidelines–Resuscitation (GWTG-R) program did not support the role of RRTs in preventing hospital mortality among 56 hospitals (5.1 million hospitalizations) from 2000 to 2014 [15]. Moreover, a systematic review reported inconsistent findings to support the role of RRTs in cardiopulmonary arrest mainly due to its various definitions in studies (eg, code blue and respiratory arrest). A meta-analysis was not conducted due to heterogeneity of the study designs (eg, data collection and sample characteristics) [16]. Randomized trials also had limited support for or did not support the role of early warning scores or RRTs in adverse events, cardiac arrest, and hospital mortality [17].

IHCA prediction has been challenging. One of the most important barriers is lack of publicly available prearrest data [18], in particular diagnostic tests, treatments (eg, medications), and physiological measures (eg, laboratory test results). The GWTG-R program of the American Heart Association [19] is a national registry that collects resuscitation data from approximately 20% of all hospitals across the country. The quality of care has improved for patients with IHCA by enhancing resuscitation guidelines and standard of care using data from the GWTG-R program. However, no longitudinal clinical data that provide an insight into the pathophysiological prearrest status (eg, laboratory test results and medications) can be found in any public cardiac arrest or resuscitation registries. Our understanding of underlying pathophysiological statuses that progress to instabilities and, ultimately, lead to cardiac arrest continues to be limited. Electronic health records (EHRs) and monitoring systems (eg, telemetry) at hospitals contain digital and time-series data that can be used to generate timely predictions. However, the presence of limited retrospective prearrest studies (eg, involving laboratory test results and medications) may indicate a complex and time-consuming process for data mining in EHRs [20-24]. Consequently, there has been no prospective study using vast digital data from hospitals to predict IHCA. Another challenge in predicting IHCA is the heterogeneity in IHCA data as the function of patients’ clinical characteristics (eg, medical-surgical diagnoses) and hospitals’ characteristics (eg, urban vs rural), particularly in methods of calculating (eg, BMI), presenting, and reporting clinical data. For example, there are different critical values for laboratory test results across health care settings [25,26]. The phenotyping approach of EHR data can be one option for some of these challenges because one size does not fit all. External validations of predictive tools can be limited without phenotyping the clinical data. Other important challenges are missing data and concerns about data accuracy. For example, vital signs recorded in the EHR are frequently used to develop clinical tools (eg, early warning scores) for predicting IHCA. However, these measures depend on bedside clinicians’ documentation, and studies have raised concerns about their accuracy and completeness [27,28].

Nevertheless, EHRs are an important source of big data for developing machine learning (ML) models to predict adverse health events. ML, a branch of artificial intelligence, uses algorithms to recognize patterns in large, diverse datasets and predict outcomes [29]. Unlike traditional statistical methods, which are limited to linear relationships between variables, ML can learn automatically to uncover complex, nonlinear relationships and aims to make the prediction as accurate as possible in massive datasets adjusting to the scale of the data without compromising the performance [30]. This makes ML well suited for IHCA prediction. The growing availability of EHR data enables the creation of predictive models that may offer more nuanced insights into the factors leading to IHCA. Despite the increasing use of ML, this field remains challenging as the quality and accuracy of EHR data are essential to ML’s success [30-34]. Because the accuracy of ML predictive models depends heavily on unbiased and diverse training data, investigators developing these models must have a comprehensive understanding of the known causes of the event or condition they are predicting, characteristics of the targeted population, and confounding variables (eg, sex differences), as well as access to a large, accurate set of time-series data.

Despite the clinical and organizational challenges to studying IHCA, such as missing or inaccurate data, the number of studies using ML models to predict IHCA has been increasing in recent years. However, there is limited IHCA review literature (eg, systematic reviews) examining the prognostic and predictive prearrest factors for IHCA. Only 1 systematic review study [35] was found that evaluated the performance of ML models in predicting IHCA by comparing them with a clinical predictive tool (ie, modified early warning score), and it concluded that ML models were superior. The quality and quantity of input data were not examined in this study; instead, the emphasis was on evaluating the performance of the ML models. Furthermore, several selected studies included composite outcomes (eg, combination of death, cardiac arrest, and unit transfer) and did not provide separate ML metrics specifically for predicting IHCA. This review was conducted in 2020 and did not include literature published in the past 4 years. In addition, the term “in-hospital cardiac arrest” was not used as one of the search keywords for finding the ML articles.

Given the increasing use of ML models in predicting IHCA and the clinical and organizational challenges associated with this emerging field, it is imperative to conduct a scoping review to identify gaps in current ML studies. These challenges include missing data and the complexities of working with EHRs in clinical settings. To the best of authors’ knowledge, no existing scoping review has synthesized and critically evaluated the quality and quantity of selected features used in ML models for predicting IHCA. This includes aspects such as patient characteristics, the temporal characteristics of collected data (eg, time of IHCA), the predictive value of prearrest clinical features, and the performance metrics of ML models. Population representativeness is essential in constructing ML models because findings then can be generalized to similar populations. Historically, women have been underrepresented in cardiovascular clinical trials, and they only represent 30% to 40% of the total population participating in sudden cardiac arrest studies [36,37]. Furthermore, there are physiological differences between men and women that are reflected in their signs and symptoms. For example, there is a higher rate of respiratory insufficiency before cardiac arrest and a lower rate of obstructed coronary artery disease after cardiac arrest in women than in men [38]. Therefore, adequate sample sizes of men and women considering their physiological differences should be considered before and during the construction of ML models (eg, training). Selection of other demographic characteristics (eg, race) should also be part of sample construction to enhance population representativeness. Another important issue in synthesizing ML studies is the presence of tools that interpret the findings and their implications in clinical practice. We will evaluate not only the quality of the data but also their relevance to clinical practice. Findings from this scoping review will not only highlight these gaps but also provide practical recommendations for standardized reporting of clinical features as integral components of ML modeling. Such standardization is crucial for improving the accuracy and applicability of ML models in clinical decision-making processes to improve the quality of patient care.

This need for standardization becomes even more evident when considering IHCA, which is a multifactorial event characterized by dynamic and complex physiological processes often involving nonlinear interactions between time-sensitive variables such as heart rate variability (HRV), oxygen saturation, and blood pressure. Traditional statistical models are typically limited by assumptions of linearity and variable independence, which constrain their ability to model such complexity. Recent work by Lee et al [39] developed and validated a real-time ML model using electrocardiogram-derived HRV metrics in intensive care unit patients and demonstrated that ML models can effectively capture nonlinear and time-dependent patterns in prearrest physiology. Their model, which used a gradient boosting algorithm, achieved an area under the receiver operating characteristic curve of 0.881 and significantly outperformed a traditional clinical parameter–based model (area under the receiver operating characteristic curve=0.735; *P*<.001), underscoring the advantage of ML in managing IHCA prediction complexity [39]. In addition, the same study highlights how ML models incorporate nonlinear HRV measures (eg, triangular interpolation of the NN interval, HRV triangular index, and inverse of average length of the acceleration and deceleration segments), which have shown predictive value but are often not feasible to model using standard regression approaches. Furthermore, evidence from Akintoye et al [40] demonstrates a nonlinear association between hospital cardiopulmonary resuscitation volume and IHCA survival, where survival peaked at a moderate volume threshold and declined at higher volumes, suggesting complex institutional and systemic interactions that ML models are well suited to accommodate hospital volume and cardiopulmonary resuscitation. These findings collectively strengthen the appropriateness of ML approaches in this context and support our rationale for focusing the scoping review on ML-based IHCA prediction models.

## Methods

This scoping review will be conducted using the PRISMA (Preferred Reporting Items for Systematic Reviews and Meta-Analyses) guidelines developed using checklists from the PRISMA-ScR (Preferred Reporting Items for Systematic Reviews and Meta-Analyses extension for Scoping Reviews), and the protocol was developed following the PRISMA-P (Preferred Reporting Items for Systematic Reviews and Meta-Analyses Protocols) [41,42].

### Review Questions

Our review questions are as follows:

1. What are the quality and quantity characteristics of clinical data used to construct ML models?
2. What are the common clinical predictors among multiple ML models?
3. What are the strongest predictive metrics for occurrence of IHCA?

### Ethical Considerations

This review will not involve direct contact with human participants. All clinical data (ie, laboratory test results and vital signs) used in ML model construction will be presented as aggregated and deidentified data from previously published studies. This protocol was registered in the Open Science Framework on November 16, 2024 [43].

### Eligibility Criteria

The focus of this review is to synthesize the clinical features used to construct ML models and evaluate the performance of these models in predicting IHCA.

The inclusion and exclusion criteria are as follows. We will include only peer-reviewed, English-language studies published between April 2009 and April 2024 that used ML algorithms to predict IHCA. The selection of 2009 as the starting year for our search was based on significant advancements in ML applications within health care around that time. In 2009, a prominent national report indicated that only a small proportion of US hospitals had adopted a “basic” EHR system [44]. In addition, the Health Information Technology for Economic and Clinical Health Act provided incentives for health care providers who used EHRs. This period marked the beginning of more widespread adoption of EHRs and the integration of advanced computational methods, which facilitated the application of ML algorithms in clinical settings. These technological advancements enabled more accurate and efficient prediction models for IHCA. Therefore, studies published after 2009 are more likely to reflect the current state of ML applications in predicting IHCA [45,46]. We will exclude the following types of articles:

1. Reviews (eg, systematic reviews and meta-analyses)—while review articles will be excluded from the formal selection, we will use them as supplementary resources to identify additional relevant studies that may not have been captured in our initial search, which will allow us to ensure a more comprehensive inclusion of relevant literature
2. Preprints, conference proceedings, theses, and dissertations
3. Studies published in foreign languages, abstract-only studies, opinions, letters to the editor, commentaries, short communications, and patents

Regarding the patient population, only studies involving adults aged ≥18 years who have experienced IHCA will be included. There will be no restriction based on sex, gender, geographic location, race, or types of ML techniques used. We will use the Utstein Out-of-Hospital Cardiac Arrest Resuscitation Registry Template definition of IHCA [1], which refers to the delivery of defibrillator shocks or chest compressions to a patient admitted to an inpatient bed. Studies that do not adhere to this definition or that did not collect data from hospitals or medical centers will be excluded. In addition, studies that include cases of death or sudden death without resuscitation or OHCA and animal studies will be excluded.

Regarding study outcomes and ML metrics, some studies list composite outcomes, including IHCA, admission to intensive care units, and patient deterioration. We will include studies that report ML metrics for predicting IHCA, even when they are part of a composite outcome, as long as the prediction of IHCA is clearly specified and distinguishable from other outcomes. This approach will allow us to capture a broader range of clinically relevant studies while maintaining focus on IHCA prediction.

### Information Sources

Four reputable databases were searched: PubMed (biomedical research), Web of Science (a global citation database in the sciences, arts, and humanities), IEEE Xplore (for technology and engineering research), and Embase (medical research). The following 2 sets of search terms—“in-hospital cardiac arrest” and “machine learning”—were entered into the databases connected using AND after several trials to select different text words.

### Search Strategy

Before protocol registration on the Open Science Framework in November 2024, we conducted a preliminary exploratory search to refine the search terms, databases, and feasibility of conducting this review. While this process involved screening a small set of articles for pilot-testing purposes, the formal and comprehensive literature search used for study inclusion and data extraction was carried out after protocol registration. This preregistration phase did not influence the core research objectives, inclusion and exclusion criteria, or data extraction domains. An interprofessional informationist team member worked with disciplinary faculty and created the base search to refine key concepts. The informationist completed a pilot search in PubMed, which a team member screened to determine retrieval accuracy for key articles on IHCA and ML. For example, we initially selected the following search terms and keywords using the MeSH (Medical Subject Headings) database:

*(“In-hospital cardiac arrest” [text word] OR “In-hospital cardiac arrest” [tiab] OR “cardiopulmonary resuscitation” [text word] OR “heart arrest” [text word] OR “sudden cardiac death” [text word] OR asystole [text word] OR heart arrest [MeSH] OR asystole [MeSH] OR cardiopulmonary arrest [MeSH] OR arrest, cardiopulmonary [MeSH]) AND* *(“Artificial intelligence” [text word] OR “artificial intelligence” [tiab] OR Artificial intelligence [MeSH] OR Intelligence, Artificial [MeSH] OR Computational Intelligence [MeSH] OR Intelligence, Computational [MeSH] OR Machine Intelligence [MeSH] OR Machine Learning [MeSH] OR Deep Learning [MeSH] OR Supervised Machine Learning + [MeSH] OR Unsupervised Machine Learning [MeSH] ) 2009-2024*

Each trial was conducted using carefully selected keywords involving Boolean operators and MeSH options along with other text words with or without the assigned years (2009-2024), which produced a set number of articles. These articles were reviewed by a team member with expertise in IHCA and familiarity with ML techniques. A discussion was then initiated between the informationist and the team member to improve search terms for targeted peer-reviewed articles in PubMed. This process was iterated 7 times, producing a range of published articles from 95 to 2004. On the basis of the search sensitivity, revisions were made to the search query, which was then translated from PubMed into 3 additional databases—Web of Science, Embase, and IEEE Xplore—for hand searching. A second research librarian will conduct a full Peer Review of Electronic Search Strategies review of the search and will recommend revisions.

### Screening

All search results will be downloaded and exported into Rayyan (Qatar Computing Research Institute) for deduplication. Automated tools in Rayyan will be used to identify and remove duplicates. Once duplicates are removed, the titles and abstracts will be screened according to the exclusion criteria, and the articles that do not meet the criteria will be excluded. The full texts of the remaining articles will then be reviewed to determine eligibility, with reasons for exclusion recorded and reported in the final scoping review. Two reviewers will independently conduct the screening process using the Rayyan application. Any disagreement between the reviewers will be resolved through discussion, with input from experts in resuscitation and data sciences. The results of the search (ie, the included articles) will be presented in a PRISMA flow diagram.

### Data Extraction and Main Data Elements

**Figure 1:**
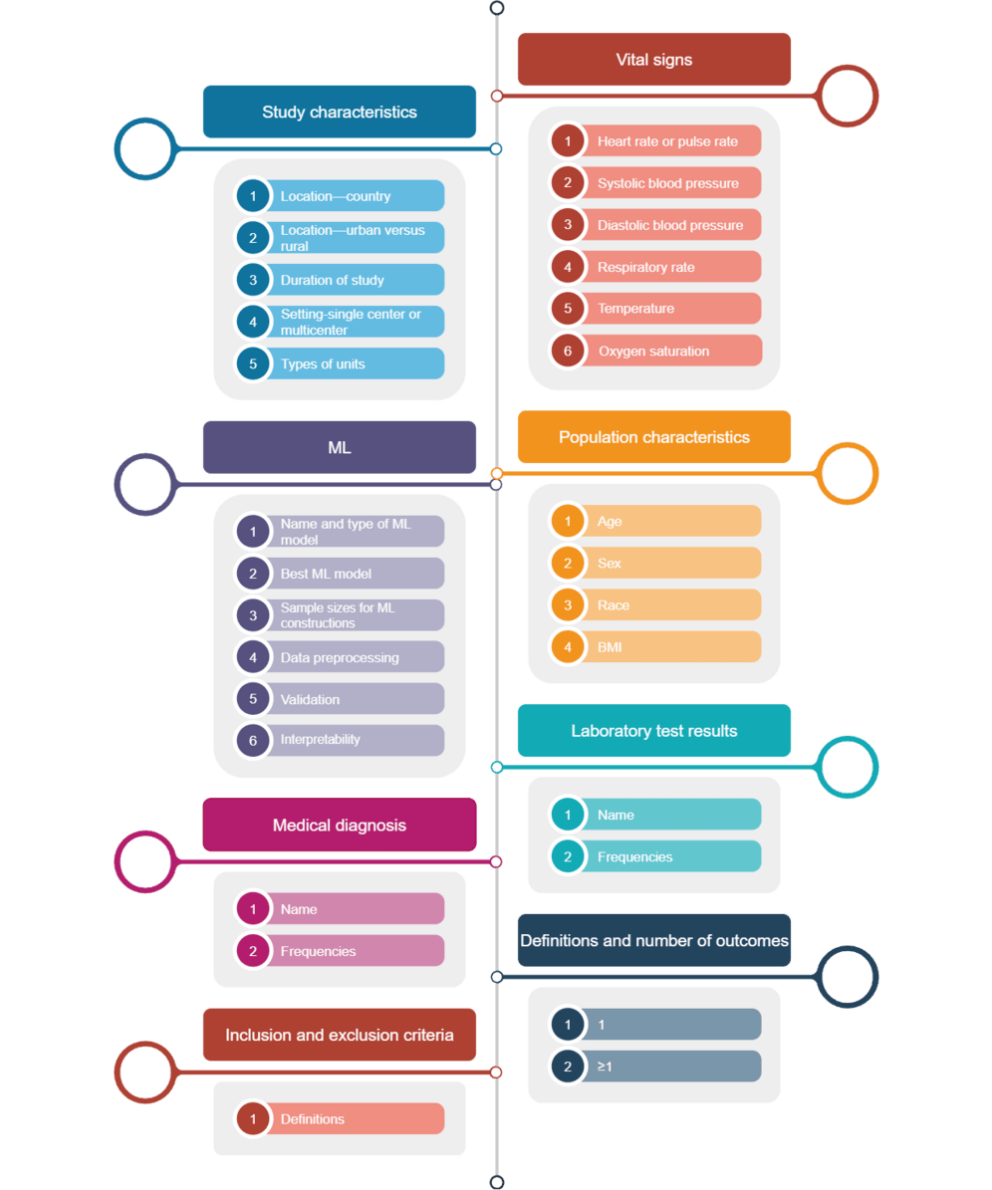
Names of the variables that will be extracted from the selected articles. ML: machine learning.

Our review aims and questions will guide the types of data elements for extraction. The targeted data elements will be identified and extracted from the eligible full texts of the articles by 2 team members who will record them as variables in a developed template using Microsoft Excel. We will have several sheets within a Microsoft Excel file to complete data charting (eg, demographics and biomarkers). The Microsoft Excel file can be considered our tool and will be revised as necessary during the extraction phase. The extracted data will be reviewed by the third team member for accuracy. To resolve any conflict, a discussion among all these team members will take place to reach consensus. A list of data elements for future extraction is presented in Figure 1, which includes study characteristics, ML metrics, medical diagnosis, inclusion and exclusion criteria, vital signs, population characteristics, laboratory test results, and definitions and number of outcomes. We specifically pay attention to 2 important criteria, including representativeness of the population by examining the sample sizes in subgroups (eg, men and women) of the total population and in the construction of ML models (eg, training and testing phases). The physiological differences between men and women that are represented by their signs and symptoms will also be evaluated. We will also determine the interpretability of the clinical data through the utility of different tools, including Shapley additive explanations.

The vital sign metrics comprise heart rate or pulse rate, systolic blood pressure, diastolic blood pressure, respiratory rate, temperature, and oxygen saturation. These metrics were selected after reviewing the literature [27,47,48] and a discussion among 2 investigators in this scoping review with medical knowledge and expertise (MA and KB). For example, oxygen saturation is typically not included in its traditional format [49], or certain vital sign metrics were not selected in some studies (eg, diastolic blood pressure) [47,48]. We decided to incorporate all vital sign metrics to comprehensively evaluate the quality and quantity of the clinical data.

### Data Analyses and Presentation

**Figure 2:**
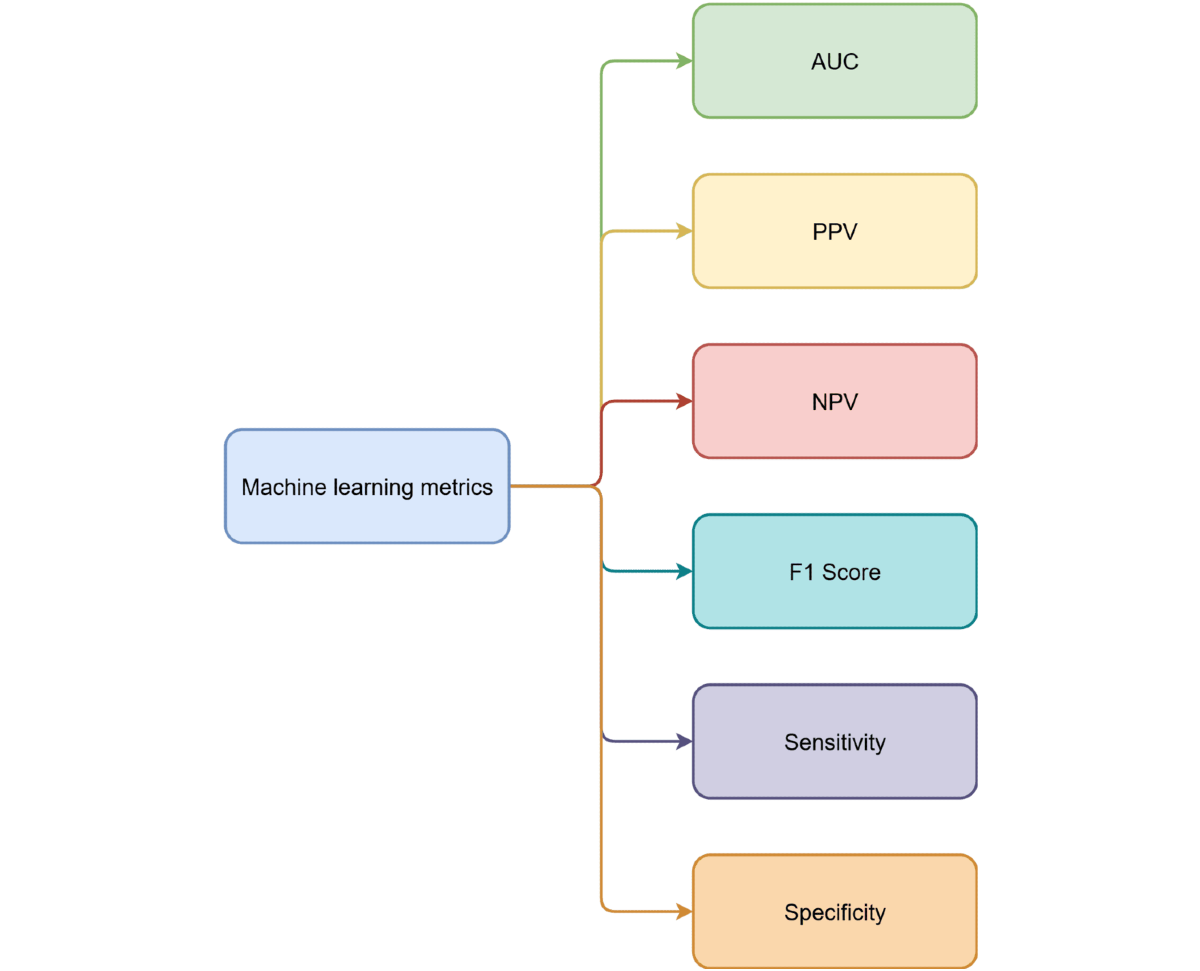
Performance metrics of machine learning models. AUC: area under the curve; NPV: negative predictive value; PPV: positive predictive value.

Descriptive statistics, including numbers, percentages, means, and SDs, will be reported to quantify the selected variables, and a comprehensive narrative will provide a detailed synthesis of variables across the articles. We will extract clinical features used in ML models for predicting IHCA, including patient demographics (eg, age and sex) and medical histories (eg, diagnoses), vital signs, laboratory test results, and all other available data. We will also gather information regarding the data sources (eg, EHRs and registries) and the methods used to handle missing or incomplete data. We will evaluate the number of features included in the models and the sample sizes used for constructing ML models. The number and type of all ML models (ie, traditional vs deep neural network) will be presented in a graph. The metrics of ML model performance will be presented in a table including the area under the curve, sensitivity, specificity, negative predictive value, positive predictive value, accuracy, precision, and *F*_1_-score (Figure 2). We will compare the performance metrics of different ML models to understand how data quality influences model outcomes. Subgroup analyses will also be conducted to evaluate the effects of different types of clinical data (eg, vital signs vs laboratory test results) on predictive performance. To ensure alignment with our review objectives, the synthesis of extracted data will be directly mapped to the review questions. We will group and summarize findings on the geographical distribution of studies, data quality and quantity, shared clinical predictors, and performance metrics using descriptive statistics (eg, frequency, percentages, means, and SDs, described previously) and a narrative approach. This dual synthesis strategy will allow us to highlight patterns in ML model inputs and outputs, methodological heterogeneity, and gaps in reporting practices. Figure 1 outlines the core variables identified for extraction and synthesis, reinforcing the linkage between data elements and our research questions.

## Results

A total of 2479 records were identified from 4 databases (Embase, PubMed, Web of Science, and IEEE Xplore). After removing 20.61% (511/2479) duplicates, 1968 titles and abstracts were screened. Of these 1968 titles and abstracts, 83 (4.22%) full-text articles were assessed for eligibility, and 16 (19%) studies have been included in the final synthesis. Data extraction and analysis are ongoing and expected to be completed by June 2025. We will present our work in national meetings relevant to the topic of cardiac arrest and resuscitation and submit our findings to a peer-reviewed journal to share knowledge and increase the awareness of the state of the art in predicting IHCA using artificial intelligence.

**Figure 3:**
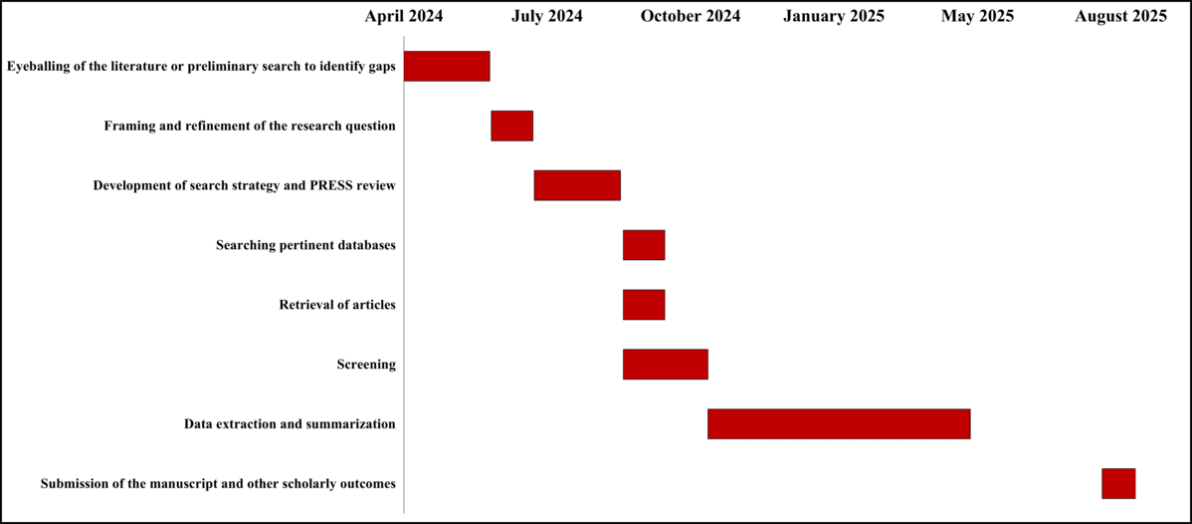
Gantt graph showing the project milestones and associated timelines. PRESS: Peer Review of Electronic Search Strategies.

The results will address each of our questions by providing specific findings presented as descriptive statistics and a detailed narrative summary. We created a comprehensive search strategy to identify studies that used ML models to predict IHCA. Our work will be presented as a scoping review that contains detailed and aggregated information summarizing data using tables and graphs. Figure 3 shows project milestones and associated timelines.

## Discussion

### Anticipated Findings

We will conduct a comprehensive evaluation of the clinical features extracted from EHRs for each phase of ML model development, including training, validation, and testing. This evaluation will focus on assessing population diversity, such as age, sex, race and ethnicity, and comorbidities, to ensure inclusivity and representation in model development. Recognizing that EHR data often contain time-series information, we will thoroughly analyze the temporal characteristics of clinical features, particularly in relation to the timing of IHCA onset. This includes exploring patterns, trends, and critical time points that could enhance the predictive power of ML models.

Furthermore, we will identify and discuss the significance of commonly reported predictors across studies, such as vital signs, laboratory test results, and comorbid conditions, and critically evaluate their clinical implications. This will provide insights into the relevance of these predictors in the context of IHCA and their potential utility in real-world clinical settings.

Special emphasis will be placed on enhancing the interpretability of ML models, addressing the *black box* challenge often associated with these approaches. Techniques such as feature importance analysis, visualization of decision pathways, and integration of domain knowledge will be explored to improve the transparency and usability of ML predictions. The discussion will also cover the metrics and methodologies for optimizing model performance, including sensitivity, specificity, precision, recall, and calibration, with a focus on their implications for clinical decision-making and patient outcomes. Through this synthesis, we aim to support the standardization of reported clinical features and methodologies in the development of predictive ML models for IHCA. This standardization will facilitate comparability across studies; enhance reproducibility; and, ultimately, contribute to the broader adoption of ML tools in clinical practice for early detection and prevention of IHCA.

### Strengths and Limitations

This study will have several strengths and limitations. Among its strengths, it will provide a comprehensive analysis of clinical features extracted from EHR data considering population diversity and temporal dynamics. This ensures that the findings are inclusive and applicable across varied patient populations. The focus on temporal characteristics is another strength as it addresses the critical aspect of timing in predicting IHCA, enabling better anticipation of adverse events. In addition, this study places significant emphasis on interpretability, tackling the *black box* nature of ML models and ensuring that results are transparent and usable by clinicians, fostering trust and adoption in health care. Furthermore, the synthesis of predictors and features across studies will support standardization in reporting practices, enhancing reproducibility and comparability in ML model development. The emphasis on clinical relevance will bridge the gap between technical advancements and practical implementation, whereas considerations of population diversity will promote equity and reduce biases in predictive models.

However, this study will also face certain limitations. The quality, completeness, and standardization of EHR data, which can vary across institutions, may impact the analysis and model performance. Despite efforts to include diverse populations, biases in available datasets could limit the generalizability of findings, especially for underrepresented groups. While interpretability is emphasized, some ML models may remain opaque due to their inherent complexity. In addition, a scoping review has inherent limitations that stem from its methodology and objectives, which focus on mapping existing literature rather than providing definitive answers to specific research questions. One primary limitation is the lack of depth in analysis as scoping reviews typically do not involve a critical appraisal of the quality of the included studies. This means that studies with varying levels of rigor may be included, potentially affecting the reliability of the findings. In addition, the heterogeneity of evidence, including diverse study designs, populations, and outcomes, can make it challenging to synthesize findings into cohesive conclusions. Unlike meta-analysis, scoping reviews do not perform formal meta-analyses or provide pooled effect estimates, limiting their ability to quantify relationships or provide definitive conclusions. The broad scope of this scoping review may also introduce selection bias as the inclusion of studies depends on the search strategy, availability of data, and reviewer judgment. Moreover, this scoping review will rely heavily on published and accessible literature, potentially overlooking unpublished studies, gray literature, or ongoing research, leading to publication bias. Despite these challenges, this study provides valuable insights and a balanced contribution to the development and evaluation of ML models for predicting IHCA.

### Conclusions

Our scoping review will add to the body of IHCA literature by synthesizing different aspects of ML studies to predict IHCA. This will be our first step to comprehend the complexity of ML studies and promote realistic options for the clinical decision-making process.

## Appendix

Multimedia Appendix 1 PRISMA-P checklist.

Multimedia Appendix 2 PRISMA-ScR checklist.

## Abbreviations

EHR: electronic health record
GWTG-R: Get With the Guidelines–Resuscitation
HRV: heart rate variability
IHCA: in-hospital cardiac arrest
MeSH: Medical Subject Headings
ML: machine learning
OHCA: out-of-hospital cardiac arrest
PRISMA: Preferred Reporting Items for Systematic Reviews and Meta-Analyses
PRISMA-P: Preferred Reporting Items for Systematic Reviews and Meta-Analyses Protocols
PRISMA-ScR: Preferred Reporting Items for Systematic Reviews and Meta-Analyses extension for Scoping Reviews
RRT: rapid response team

## Data Availability

The datasets generated or analyzed during this study are available from the corresponding author on reasonable request.
